# Five-year predictors of physical activity decline among adults in low-income communities: a prospective study

**DOI:** 10.1186/1479-5868-4-2

**Published:** 2007-01-18

**Authors:** Deborah R Weiss, Jennifer L O'Loughlin, Robert W Platt, Gilles Paradis

**Affiliations:** 1 Centre for Clinical Epidemiology and Community Studies, Jewish General Hospital, Montréal, Québec, Canada; 2 Department of Social and Preventive Medicine, Centre de recherche CHUM, Université de Montréal, Montreal, Québec, Canada; 3 Department of Epidemiology, Biostatistics and Occupational Health, Faculty of Medicine, McGill University, Montreal, Québec, Canada; 4 Montreal Children's Hospital Research Institute, Montreal, Québec, Canada; 5 Department of Pediatrics, McGill University, Montreal, Québec, Canada; 6 Institut national de santé publique du Québec Montreal, Québec, Canada

## Abstract

**Background:**

Obesity in North America is now endemic, and increased understanding of the determinants of physical inactivity is critical. This analysis identified predictors of declines in physical activity over 5 years among adults in low-income, inner-city neighbourhoods.

**Methods:**

Data on leisure time physical activity were collected in telephone interviews in 1992 and 1997 from 765 adults (47% of baseline respondents), as part of the evaluation of a community-based cardiovascular disease risk reduction program.

**Results:**

One-third of 527 participants who were physically active at baseline, were inactive in 1997. Predictors of becoming inactive included female sex (OR = 1.63 95% CI (1.09, 2.43)), older age (1.02 (1.01, 1.04)), higher BMI (1.57 (1.03, 2.40)), poor self-rated health (1.39 (1.05, 1.84)), lower self-efficacy for physical activity (1.46 (1.00, 2.14)), and not using a neighborhood facility for physical activity (1.61 (1.02, 2.14)).

**Conclusion:**

These results highlight the fact that a variety of variables play a role in determining activity level, from demographic variables such as age and sex, to psychosocial and environmental variables. In addition, these results highlight the important role that other health-related variables may play in predicting physical activity level, in particular the observed association between baseline BMI and the increased risk of becoming inactive over time. Lastly, these results demonstrate the need for multi-component interventions in low-income communities, which target a range of issues, from psychosocial factors, to features of the physical environment.

## Background

Physical inactivity is a major risk factor for obesity, coronary heart disease, type 2 diabetes, certain types of cancer, and osteoporosis [1-5]. Sixty percent of American adults [6], and 62% of Canadian adults [7], are not sufficiently active to attain health benefit from physical activity. Estimates indicate that 25% of American adults engage in no physical activity at all [6]. Increased understanding of the reasons why people become and remain inactive is essential to design more effective public health programs to reduce health problems related to inactivity, especially in disadvantaged communities, which have higher levels of inactivity [8,9], an excess burden of disease associated with inactivity [8,10-13], and in which community-based efforts to improve physical activity have had little success [14].

Determinants of the adoption and maintenance of healthy physical activity behaviors in adults identified to date include demographic factors (age [10,11,15,16], sex [6], socio-economic status (SES) [8,10,11,13]), psychosocial factors (social support, self-efficacy, perceived barriers [17-22]), and features of the physical environment (i.e., access to sports facilities and neighbourhood safety [23-28]). However few longitudinal studies identify predictors of declines in physical activity [20-22,29-31] and only one of these longitudinal studies was conducted in low SES communities [22]. Longitudinal studies are essential so that a temporal relationship between a putative predictor and physical activity can be established.

To identify predictors of declines in physical activity levels among socio-economically disadvantaged adults, a secondary analysis was conducted of data collected as part of the evaluation of Cœur en santé St. Henri, a community-based cardiovascular disease (CVD) risk reduction program targeting adults in a low-income, inner-city neighbourhood in Montreal, Canada [32-34].

## Methods

Cœur en santé St. Henri was a 4-year (1992–5) community-based CVD risk reduction program targeting adults aged 18–65 years living in St. Henri, a low-income, low-education neighborhood in southwest Montreal, Canada [32]. The objectives of the intervention program were to promote heart-healthy behaviours, including a low-fat diet, non-smoking, increased physical activity as well as blood pressure and cholesterol control. During the 48-month implementation phase, more than 40 interventions were implemented, including smoking cessation workshops, contests, newspaper columns and advertising, nutrition workshops, menu labeling in local restaurants, as well as direct-mail programs to promote healthy weight regulation. The impact of the program was evaluated in a quasi-experimental study design that compared levels of modifiable CVD risk factors among adults in St. Henri to those of adults in a matched comparison community. Study participants were randomly selected from within households that were randomly selected within the two study communities, in a two-stage cluster sampling design. Households in which there were no eligible persons in the age range of interest and those in which potential participants did not speak either French or English, were excluded. Data were collected in 35-minute telephone interviews at baseline in 1992 and again, from the same participants, in 1997. Detailed descriptions of the study design and methods have been reported [33,34]. Because the program had no impact on levels of physical activity [33] data from the intervention and comparison communities were pooled in the current analysis.

### Study variables

Frequency of Leisure Time Physical Activity (LTPA) was assessed in two questions adapted from the previously validated Leisure-Time Exercise Questionnaire [35], including: (i) "Think back over the past three months. In a typical week, how many hours do you spend in vigorous leisure time physical activity which causes you to perspire and breathe hard?" and (ii) "In a typical week, how many hours do you spend in moderate leisure time physical activity, such as brisk walking, bicycling, or heavy gardening?" Responses were summed to create a continuous LTPA score. The Leisure-Time Exercise Questionnaire has been found to be both valid and reproducible [36]. Because it is widely recommended that adults engage in physical activity at least 5 days per week, 30 minutes per session (i.e. 150 minutes per week) in order to maintain good health [6], we dichotomized "LTPA status" into "active" (150 minutes or more of activity each week), and "inactive" (up to 150 minutes per week).

Body mass index (BMI), computed as weight (kg)/height(m^2^) based on self-reported height and weight, was dichotomized into less than 25, and greater than or equal to 25 [36,37]. All subjects included in this study answered the questions about height and weight. A smoking status variable was created, with three categories: (i) current smoker (has ever been a daily smoker, and has not quit smoking permanently); (ii) past smoker (has ever been a daily smoker and has quit smoking permanently); and (iii) never smoker. Self-rated health was assessed by; "In general, compared to other persons your age would you say your health is...(excellent; good; average; poor/very poor)".

Social support for physical activity was measured in one item: "Is there anyone encouraging you to be physically active?" (yes; no). Self-efficacy related to physical activity was assessed in three items: "Tell me if, for you, the following would be easy (scored 1), somewhat difficult (scored 2) or very difficult (scored 3)...(i) to exercise even when you feel like doing something else (ii) to organize yourself to exercise regularly (iii) to try new kinds of physical activity." Summing scores across the three items generated an indicator of self-efficacy with a reliability coefficient of 0.58, and with higher scores indicating lower self-efficacy.

Use of a neighborhood facility for physical activity was determined by; "During the last year, did you use any of the centers for physical activity in your neighborhood (for exercising); yes or no?"

### Data analysis

The outcome of interest was LTPA status at follow-up among participants who were active at baseline. It was assumed that the LTPA status among participants who were active at baseline and inactive at follow-up had declined over the five-year follow-up period.

Potential predictors of decline in LTPA status investigated included sociodemographic indicators (i.e., sex, age, income, level of education), psychosocial variables (i.e., social support, self-efficacy), self-reported health status, BMI, cigarette use, and use of neighborhood facilities for exercise.

Potential predictors of decline in LTPA status significant at p < 0.1 in univariate analyses were retained for multivariate logistic regression analysis. Variables were retained in the final model if they were significant at p < 0.05. Variables not retained were entered into the final model one by one to check for confounding (i.e., if their inclusion affected the point estimates of other variables by 10% or more). All statistical analyses were conducted using SAS v 8.2 (SAS Institute Inc., Cary, NC, 1999).

## Results

A total of 765 participants (47% of 1674 participants recruited into the evaluation study) were followed up in 1997. Participants lost to follow up were younger, more were male, and more had completed high school [33]. Of 765 participants followed up in 1997, 238 were excluded from this analysis, including 107 who were inactive at baseline and therefore not at risk of becoming inactive at follow-up, and 131 with incomplete data on physical activity. A total of 527 participants who were active at baseline and who had complete follow-up data comprised the study population.

The mean age of the 527 participants was 37 (SD = 11) years (range = 18–65) at baseline, 47% were male, 40% had some post-secondary education, and almost three-quarters were Francophone. The median LTPA score among all participants at baseline was 7.5 hours/week (range = 2.5–51.0), and 4.0 hours/week (range = 0–40.0) at follow-up. The median LTPA score among the 334 participants who remained active at follow-up was 8.0 hours/week (range = 2.5–51.0) at baseline, and 7.0 (range = 3.0–40.0) hours/week at follow-up. The median LTPA score among the 193 participants who became inactive was 7.0 hours/week (range = 2.5–42.0) at baseline, and 0.0 (range = 0–2) hours/week at follow-up.

In 1997, 36% of participants (n = 193 of 527) had become inactive, including 31% of males and 42% of females. Variables univariately associated with decline in LTPA status included female sex, older age, lower levels of education, poor self-rated health, lower self-efficacy, higher BMI, and not using a neighbourhood facility for physical activity (Table 1). Older age, female sex, being overweight, lower self-efficacy, poor self-rated health, and not using a neighborhood facility for activity were retained in the multivariate model (Table 1). In addition, cigarette use and income were retained because their inclusion resulted in a greater than 10% change in the odds ratio for the self-efficacy variable. None of the interaction terms tested for sex attained statistical significance.

**Table 1:** Unadjusted and adjusted Odds Ratios for potential predictors of becoming inactive

Potential predictor	Participants who became inactive	Unadjusted OR (95% CI)	Adjusted OR (95% CI)
Sex, % (n)			
Male	31.1 (77)	1.00 (Ref.)	1.00 (Ref.)
Female	41.6 (116)	1.58 (1.10, 2.27)	1.63 (1.09, 2.43)
Age (years), mean (SD)	39.5 (12.2)	1.03 (1.01, 1.05)	1.02 (1.01, 1.04)
Self-rated health, % (n)			
Excellent	29.8 (50)	1.70 (1.35, 2.16)	1.39 (1.05, 1.84)
Good	32.6 (84)		
Average	57.8 (48)		
Poor/Very poor	64.7 (11)		
BMI^a^, % (n)			
<25	32.1 (117)	1.00 (Ref.)	1.00 (Ref.)
≥ 25	46.6 (76)	1.84 (1.26, 2.69)	1.57 (1.03, 2.40)
Smoking status, % (n)			
Current smoker	37.5 (77)	1.00 (Ref.)	1.00 (Ref.)
Past/Never smoker	36.2 (121)	1.06 (0.73, 1.53)	0.98 (0.78, 1.23)
Income, % (n)			
<20,000	39.5 (68)	0.82 (0.65, 1.03)	0.95 (0.73, 1.24)
20,000–40,000	35.8 (62)		
40,000+	30.2 (42)		
Self-efficacy score, mean (SD)	2.2 (0.6)	1.61 (1.20, 2.26)	1.46 (1.00, 2.14)
Use of a neighborhood facility for activity, % (n)			
No	41.3 (152)	1.00 (Ref.)	1.00 (Ref.)
Yes	25.8 (41)	2.03 (1.34, 3.06)	1.61 (1.02, 2.55)
Education, % (n)			
Elementary/some secondary	52.6 (50)	0.74 (0.63, 0.86)	N/A*
Completed secondary/some college	40.2 (47)		
Completed college	32.5 (27)		
Some post-secondary	29.8 (67)		
Receives encouragement for activity, % (n)			
No	35.2 (113)	1.00 (Ref.)	N/A*
Yes	38.5 (79)	0.87 (0.60, 1.25)	

^a^Body Mass Index

* Not included in final model

We conducted a sensitivity analysis in which LTPA scores were dichotomized at 90 rather than at 150 minutes/week, with no substantive differences in the results (data not shown). Finally we included baseline LTPA scores in the model. While significantly associated with being inactive at follow-up (OR = 0.96 95% CI (0.93, 0.99)), there were no substantial changes in the odds ratios for the other predictor variables, and therefore, baseline physical activity was not included in the final model (data not shown).

## Discussion

According to data from the 1999 National Population Health Survey, 62% of Canadian adults are inactive [7]. In contrast, only 17% of participants in the current study were inactive during their leisure time at baseline and 41% were inactive at follow-up. This difference likely relates to the use of differing methods of measuring physical activity, as well as differing cut-offs that were used to categorize participants as being inactive during leisure time. In the Canadian study, those expending less than 1.5 Kcal/kg/day in leisure time physical activity were said to be inactive [7]. A study published by the US Department of Health and Human Services, which used cut-offs similar to those used in this study, reported that the prevalence of physical inactivity ranged from 24% to 29% in adults [6].

This analysis provides longitudinal evidence that a variety of diverse factors predict declines in LTPA in low-income communities, and that these factors are at least qualitatively similar to those identified in other more advantaged population groups [15-21,24]. Similar to other reports, women were more likely than men to become inactive. Casperson et al. [16] reported that, while males experience greater declines in physical activity levels during adolescence, women report lower levels of activity throughout adulthood.

Older age increased the odds of becoming inactive by approximately 2% per year. Previous studies report that the greatest declines in activity levels occur during adolescence, with inactivity increasing more slowly with increasing age throughout adulthood [15,16]. Sallis [15] hypothesized that age-related declines in activity levels may be, at least in part, biologically based since declines with increasing age are observed across diverse populations, as well as in animal models.

Results regarding the association between self-rated health and physical activity are inconsistent across studies. In this current analysis, poorer self-rated health predicted physical activity declines. Our results concur with those of a previous study, which reported that less than "excellent" self-rated health was associated with a 9-year decline in physical activity, in both men and women [31].

The effect of self-efficacy observed in our analysis is supported by previous research [20,21,39]. In a 2-year follow up of men and women in the U.S., baseline self-efficacy and change in self-efficacy predicted the adoption of a physically active lifestyle in persons who were initially sedentary [20]. In contrast to previous studies, [17-19,39] social support in our study was not associated with declines in physical activity. This variable might be time-dependent and if social support changed over time, the 5-year period between questionnaire administrations in this study might have been too long to capture its effect [21].

Perhaps the most important finding of this analysis is that higher BMI predicted declines in levels of LTPA. While physical inactivity has been shown to be associated with increased BMI [40-42] our results suggest that the association may be bi-directional, such that activity levels may decline to a greater extent over time among persons who are overweight. Relative to participants who were not overweight, those who were overweight at baseline were 1.6 times more likely to be inactive at follow-up. This finding is not consistent with studies that reported no association between baseline BMI and subsequent physical activity [40,20,22]. However two of these studies [20,22] were limited by short follow-up periods of one and two years, respectively. The influence of obesity on physical activity may manifest only over relatively longer periods of time, so that studies of short duration may not capture this relationship. In addition, one study [20] assessed only vigorous physical activity. Persons who are overweight may prefer low or moderate intensity activity because of the greater energy required to mobilize body mass for vigorous activity, and because of increased osteoarthritis and low back pain [37,43]. By focusing on vigorous exercisers only, this study may have inadvertently excluded overweight or obese individuals from the study sample at baseline. Lazarus et al. [30] reported an association between baseline BMI and decreases in physical activity levels after 9 years of follow-up [30], but only among women in the heaviest BMI category, and Schmitz et al. [29] reported that an increase in BMI over 2 years, but not baseline BMI was associated with decreases in physical activity.

Physiologic, genetic and psychosocial factors might all contribute to the relationship between BMI and declines in physical activity. Overweight individuals are at increased risk of orthopaedic conditions, CVD, and type II diabetes [37,42] which might reduce their ability to exercise. Twin studies indicate that genotype influences both physical activity behavior and the tendency to store excess calories as fat [41,42]. However, no specific genes involved in both processes have been discovered to date. Psychosocial factors might play a role in the relationship between BMI and physical activity. In addition to intolerance experienced by overweight persons in social settings, at work, at school and in the health-care system [42] they may also encounter intolerance in physical activity settings. Differences in body size are more evident in clothing used for sports and exercising, and might inhibit participation in physical activity, and equipment commonly used in athletic settings might not be suitable for use by overweight individuals. In addition, it is possible that BMI acts as an effect modifier in the relationship between other variables and physical activity. For example, while the relationships were not statistically significant, it is possible that poor self-rated health is a far stronger predictor of becoming inactive in overweight and obese individuals as compared to those of a normal weight (data not shown). Future studies should investigate the complex relationship between BMI and physical activity further, with special attention to the possibility of effect modification.

Although numerous studies suggest that features of the physical environment affect physical activity levels [18,27,44,45], few longitudinal studies have explored this relationship. In one prospective study, the presence of home exercise equipment, the neighbourhood environment, and access to facilities for engaging in physical activity, predicted adoption of physical activity in initially sedentary men, over a 24-month period [20]. However only vigorous physical activity was assessed, and there was an over-representation of affluent and well-educated persons, and an under-representation of minorities, in the study sample. The results reported here, that not using a neighbourhood facility for exercise is a predictor of decline in physical activity, provides evidence that the physical environment is important to maintaining adequate levels of physical activity in disadvantaged communities. Increased availability and access to facilities in these communities may be key factors in designing interventions aimed at increasing physical activity.

### Limitations

The measure of physical activity was valid and reliable [35,36], but a more comprehensive measure might have reduced misclassification. However the sensitivity analysis suggests that misclassification of activity status did not bias the findings appreciably. Potential predictors of decline in LTPA available for investigation were limited to variables available in the original study. In particular, there were few measures of the physical environment and all were reported by the survey participants. While the length of follow-up is a strength, it may have resulted in some misclassification of time-dependent potential predictors. As mentioned, there were losses to follow-up between the two data collections, and those who were lost were proportionately more male, younger, and were more educated. Given that these variables were all associated with remaining active, this could have inflated the associations reported here. However, given that the differences between those lost and those retained were small, the effect, if any, would be slight. BMI was based on self-reports of height and weight, and was therefore subject to misclassification bias, although there is little reason to suspect that the misclassification was differential.

## Conclusion

This analysis supports that predictors of decline in physical activity in disadvantaged populations are similar to those in other population groups, and include female sex, older age, poorer health, lower self-efficacy for physical activity, and not using a neighborhood facility for physical activity. Perhaps of most interest, higher BMI was associated with declines in LTPA over time suggesting that BMI and physical activity may interact in a bi-directional way. Physical inactivity results in increases in BMI which in turn results in declines in activity levels. Overall these results highlight the importance of considering a range of diverse factors related to the individual as well the physical environment in designing interventions aimed at increasing or maintaining physical activity levels in low SES communities. Given that the predictors of decline in physical activity reported here for low SES communities are similar to those reported in other populations, it is possible that intervention strategies used in other communities may also work in low SES communities. However, the interventions themselves would need to be tailored to the communities in question, in order to take into account cultural and linguistic diversity.

## Statement of competing interests

The author(s) declare that they have no competing interests.

## Authors' contributions

DW conducted the review of the literature, carried out the statistical analyses, and wrote the manuscript. JO provided guidance with the statistical analyses, interpreting the results and writing the manuscript. RP provided guidance with conducting the statistical analysis and writing the manuscript. GP provided guidance with designing the research question, conducting the statistical analyses, interpreting the results, and writing the manuscript.
