# Correction: five-year predictors of physical activity decline among adults in low-income communities: a prospective study

**DOI:** 10.1186/1479-5868-4-23

**Published:** 2007-06-05

**Authors:** Deborah R Weiss, Jennifer L O'Loughlin, Robert W Platt, Gilles Paradis

**Affiliations:** 1 Centre for Clinical Epidemiology and Community Studies, Jewish General Hospital, Montréal, Québec, Canada; 2 Department of Social and Preventive Medicine, Centre de recherche CHUM, Université de Montréal, Montréal, Québec, Canada; 3 Department of Epidemiology, Biostatistics and Occupational Health, Faculty of Medicine, McGill University, Montréal, Québec, Canada; 4 Montreal Children's Hospital Research Institute, Montréal, Québec, Canada; 5 Department of Pediatrics, McGill University, Montréal, Québec, Canada; 6 Institut national de santé publique du Québec, Montreal, Québec, Canada

## Abstract

After publication it was brought to our attention that the information for one of the variables in Table 1 was incorrect (Weiss, O'Loughlin et al. *International Journal of Behavioral Nutrition and Physical Activity *2007, **4:**2). The variable in question is "Use of a neighborhood facility for activity". In the first column, the first row should read "yes", and the second row, "no". In the second column, the first row should read 25.8 (41) and the second row, 41.3 (152).

Unadjusted and adjusted Odds Ratios for potential predictors of becoming inactive [1].

^a^Body Mass Index

* Not included in final model

**Table 1:** Unadjusted and adjusted Odds Ratios for potential predictors of becoming inactive [1].

Potential predictor	Participants who became inactive	Unadjusted OR (95% CI)	Adjusted OR (95% CI)
Sex, % (n)			
Male	31.1 (77)	1.00 (Ref.)	1.00 (Ref.)
Female	41.6 (116)	1.58 (1.10, 2.27)	1.63 (1.09, 2.43)
Age (years), mean (SD)	39.5 (12.2)	1.03 (1.01, 1.05)	1.02 (1.01, 1.04)
Self-rated health, % (n)			
Excellent	29.8 (50)	1.70 (1.35, 2.16)	1.39 (1.05, 1.84)
Good	32.6 (84)		
Average	57.8 (48)		
Poor/Very poor	64.7 (11)		
BMI^a^, % (n)			
<25	32.1 (117)	1.00 (Ref.)	1.00 (Ref.)
=25	46.6 (76)	1.84 (1.26, 2.69)	1.57 (1.03, 2.40)
Smoking status, % (n)			
Current smoker	37.5 (77)	1.00 (Ref.)	1.00 (Ref.)
Past/Never smoker	36.2 (121)	1.06 (0.73, 1.53)	0.98 (0.78, 1.23)
Income, % (n)			
<20,000	39.5 (68)	0.82 (0.65, 1.03)	0.95 (0.73, 1.24)
20,000–40,000	35.8 (62)		
40,000+	30.2 (42)		
Self-efficacy score, mean (SD)	2.2 (0.6)	1.61 (1.20, 2.26)	1.46 (1.00, 2.14)
Use of a neighborhood facility for activity, % (n)			
Yes	25.8 (41)	1.00 (Ref.)	1.00 (Ref.)
No	41.3 (152)	2.03 (1.34, 3.06)	1.61 (1.02, 2.55)
Education, % (n)			
Elementary/some secondary	52.6 (50)	0.74 (0.63, 0.86)	N/A*
Completed secondary/some college	40.2 (47)		
Completed college	32.5 (27)		
Some post-secondary	29.8 (67)		
Receives encouragement for activity, % (n)			
No	35.2 (113)	1.00 (Ref.)	N/A*
Yes	38.5 (79)	0.87 (0.60, 1.25)	

^a^Body Mass Index

* Not included in final model
